# A composite peripheral blood gene expression measure as a potential diagnostic biomarker in bipolar disorder

**DOI:** 10.1038/tp.2015.110

**Published:** 2015-08-04

**Authors:** K Munkholm, L Peijs, M Vinberg, L V Kessing

**Affiliations:** 1 Psychiatric Center Copenhagen, Rigshospitalet, University of Copenhagen, Copenhagen, Denmark; 2 Centre of Inflammation and Metabolism, Rigshospitalet, University of Copenhagen, Copenhagen, Denmark; 3 Centre for Physical Activity Research, Rigshospitalet, University of Copenhagen, Copenhagen, Denmark

## Abstract

Gene expression in peripheral blood has the potential to inform on pathophysiological mechanisms and has emerged as a viable avenue for the identification of biomarkers. Here, we aimed to identify gene expression candidate genes and to explore the potential for a composite gene expression measure as a diagnostic and state biomarker in bipolar disorder. First, messenger RNA levels of 19 candidate genes were assessed in peripheral blood mononuclear cells of 37 rapid cycling bipolar disorder patients in different affective states (depression, mania and euthymia) during a 6–12-month period and in 40 age- and gender-matched healthy control subjects. Second, a composite gene expression measure was constructed in the first half study sample and independently validated in the second half of the sample. We found downregulation of *POLG* and *OGG1* expression in bipolar disorder patients compared with healthy control subjects. In patients with bipolar disorder, upregulation of *NDUFV2* was observed in a depressed state compared with a euthymic state. The composite gene expression measure for discrimination between patients and healthy control subjects on the basis of 19 genes generated an area under the receiver-operating characteristic curve of 0.81 (*P*<0.0001) in sample 1, which was replicated with a value of 0.73 (*P*<0.0001) in sample 2, corresponding with a moderately accurate test. The present findings of altered *POLG*, *OGG1* and *NDUFV2* expression point to disturbances within mitochondrial function and DNA repair mechanisms in bipolar disorder. Further, a composite gene expression measure could hold promise as a potential diagnostic biomarker.

## Introduction

Although there is growing evidence that inflammatory disturbances, altered neuroplasticity and disturbances related to mitochondrial function are associated with bipolar disorder, our understanding of the biological background for the disorder is inadequate. Further, assessment of diagnosis, symptoms and effect of treatment are limited in relying solely on subjective clinical information as there are no available laboratory tests.^1^ Identification of peripheral blood biomarkers of disease (trait) and/or disease activity (state)^2^ has the potential to both advance our understanding of core pathophysiological processes and to move clinical treatment of bipolar disorder ahead.^3^ Gene expression assessed in peripheral blood has emerged as a viable avenue for the identification of peripheral biomarkers;^4^ however, the evidence base for gene expression alterations of single genes in bipolar disorder is limited by a lack of replicated findings and methodological issues.^5^ Importantly, given the likely complex biological nature of bipolar disorder, a panel of genes rather than one single gene is more likely to constitute a useful tool.^6^ Studies investigating panels of genes have used a focused approach, investigating inflammation-related pathways^7^ or studied lymphoblastoid cell lines,^8^ which do not consider the current affective state and are subject to influences due to cell culture passaging.^9, 10^ Further, they did not consider affective state of participants^11^ and beyond a recent small study investigating both manic and euthymic states in 11 patients with bipolar disorder,^12^ within-subject alterations between affective states have not been included.

In the present study, using a longitudinal design that incorporated within-subject comparisons between affective states, we investigated the messenger RNA (mRNA) expression in peripheral blood mononuclear cells (PBMCs) of 19 genes that have been reported as candidate biomarker genes in comprehensive gene expression studies, genome-wide association studies or otherwise relate to current hypothesis regarding bipolar disorder pathophysiology. First, we aimed to assess differences in expression of candidate biomarker genes between (1) bipolar disorder patients and healthy control subjects and (2) between affective states in bipolar disorder patients. Second, in a split sample design, we investigated the potential for a composite gene expression measure to function as a clinically relevant biomarker that (1) discriminates between healthy control subjects and bipolar disorder patients and (2) discriminates between affective states in bipolar disorder patients, which was validated in an independent sample.

## Materials and methods

### Participants

#### Bipolar disorder patients

Inclusion criteria were a DSM-IV diagnosis of rapid cycling bipolar disorder, defined by the occurrence of at least four mood episodes (mania, hypomania, depression or mixed) during the preceding year in the context of bipolar disorder and age between 18 and 70 years. Exclusion criteria were current drug abuse, insufficient Danish language skills, pregnancy and significant physical illness (that is, chronic heart disease, chronic pulmonary disease, inflammatory disease, chronic infectious disease and neurodegenerative disease), determined by available case material, patients' self-report and routine blood chemistry tests. Patients were recruited during the period of June 2010 to May 2012 through referral by psychiatrists at hospitals or outpatient facilities throughout the region of Zealand, Denmark. A total of 37 bipolar disorder patients were included. Two bipolar patients declined further examination after 1 and 3 months follow-up, respectively, the remaining bipolar patients were followed for a minimum of 6 months with a mean (s.d.) follow-up period of 11.9 (3.0) months. Patients were evaluated with clinical assessments of mood and collection of blood samples upon signs of new affective episodes, which when possible, were repeated after return to a subsequent euthymic state or change to an affective episode of opposite polarity. Assessment and biochemical analysis were postponed in case of clinical signs of acute infection, allergic symptoms or other acute medical condition.

#### Healthy control subjects

Forty healthy control subjects were recruited among blood donors affiliated with the Blood Bank at Rigshospitalet, Copenhagen, Denmark. Inclusion criteria were no history of psychiatric disorder in the subjects or their first-degree relatives and age between 18 and 70 years, Exclusion criteria were identical to those applied to bipolar disorder patients. Healthy control subjects were evaluated with clinical assessments and collection of blood samples on two occasions ~3 months apart. Assessment and biochemical analysis were postponed if there were clinical signs of acute infection, any allergic symptoms or other acute medical condition. Mean (s.d.) follow-up time for the healthy control subjects was 2.9 (0.9) months.

Two bipolar disorder patients reported mild reflux esophagitis and four patients reported well-controlled hypertension. One healthy control subject reported previous treatment for gallstone and one reported intermittent symptoms of allergic rhinitis. No participants suffered from diabetes. All the participants provided written informed consent and were reimbursed for their travel expenses. The study protocol was approved by the Committee on Health Research Ethics of the Capital Region of Denmark (protocol no. H-4-2010-006). The study complied with the Declaration of Helsinki.

### Clinical assessments

All the participants were assessed by a specialist in psychiatry (KM), using standardized semi-structured interviews. The Schedules for Clinical Assessment in Neuropsychiatry interview^13^ was used for diagnostic purposes and was based on available case material, referral reports, the interview with the participant and the Hypomania Checklist (HCL-32)^14^ completed by the participant. A DSM-IV diagnosis of rapid cycling bipolar disorder was established for the patients and comorbid psychiatric illness, if present, was recorded. For healthy control subjects, absence of lifetime psychiatric morbidity was confirmed.

A clinical diagnosis according to DSM-IV, was established at each study visit concurrently with the collection of samples for laboratory analysis. Severity of depressive symptoms was assessed using the 17-item Hamilton Depression Rating Scale (HAMD-17)^15^ and manic symptoms were assessed using the Young Mania Rating Scale (YMRS),^16^ with a time period of 3 days applied.

Categories of affective states were based on clinical evaluation according to the Schedules for Clinical Assessment in Neuropsychiatry interview combined with the HAMD-17 and YMRS rating scales without applying duration criteria: euthymic (HAMD-17 and YMRS <8), depressive (HAMD-17 >7 and YMRS <8), manic/hypomanic (YMRS >7 and HAMD-17 <8) and mixed state (HAMD-17 >7 and YMRS >7).

### Candidate biomarker genes

Genes were selected for mRNA analysis on the basis of previous findings and current hypothesis related to the pathophysiology of bipolar disorder, focusing on evidence based on findings in peripheral blood:

#### Genes identified in genome-wide association studies

Ankyrin-3 (*ANK3*), calcium channel, voltage-dependent, L type, alpha 1C subunit (*CACNA1C*) and RAS guanyl releasing protein 1 (*RASGRP1*).^17^

#### Blood biomarker candidate genes identified using convergent functional genomics

Krueppel-like factor 12 (*KLF12*) and brain-derived neurotrophic factor.^18^

#### Candidate genes identified in lymphoblastoid cells

DNA polymerase subunit gamma (*POLG*), *ANK3*, *RASGRP1*.^8^

#### Candidate gene identified through proteomic analysis

Phosphoglycerate mutase 1 (*PGAM1*).^19^

#### Inflammation-related genes identified through whole-genome analysis

Phosphodiesterase type 4 (*BPDE4B*) and mitogen-activated protein kinase 6 (*MAPK6*).^7^

#### Mitochondrial function-related candidate genes

NADH dehydrogenase [ubiquinone] flavoprotein 2, mitochondrial (*NDUFV2*).^20^

#### DNA repair mechanism genes

Oxidatively generated damage to DNA has been demonstrated in bipolar disorder by the authors.^21^ Gene expression alterations related to DNA repair may thus accompany bipolar disorder. 8-Oxoguanine glycosylase (*OGG1*) and 7,8-dihydro-8-oxoguanine triphosphatase (*NUDT1*).

#### RNA-editing genes

Adenosine deaminase acting on RNA (*ADAR2*).^22^

#### Genes potentially related to the functional effects of lithium

RAC-alpha serine/threonine-protein kinase (*AKT1*)^23^ and glycogen synthase kinase 3 beta (*GSK3B*).^24^

#### Estrogen-related genes

Gender differences in bipolar disorder may be related to estrogen receptor function. Alterations could be linked with the G protein-coupled estrogen receptor 1 (*GPER1*),^25^ as well as estrogen receptor alpha (*ESR1*) and beta (*ESR2*).^26^

#### Transcription factor genes hypothesized as susceptibility genes in bipolar disorder

Transcription factor SP4 (*SP4*)^27^ and *SP1*.^28^

#### Apolipoprotein-related genes

Aberrant expression of the apolipoprotein E (*APOE*) gene has been demonstrated in postmortem brain tissue in bipolar disorder^29^ and APOE genotype is associated with risk of Alzheimer's disease^30^ and coronary heart disease,^31^ which are comorbid diseases with increased prevalence in bipolar disorder.

#### Candidate reference genes used in previous studies

*ACTB*,^8^ *ABL1*,^7^ *SDHA*.^32^

### Blood sampling, RNA preparation and reverse transcription quantitative real-time PCR

Blood samples were obtained in the fasting state between 2030 and 1030 h, after a minimum period of 15 min rest, concurrently with the clinical evaluation.

Nine milliliters of blood was drawn by venipuncture into a citrate phosphate dextrose adenine containing vacuum tube (Vacuette, Greiner Bio-One, Kremsmünster, Austria), which was kept at room temperature before and after blood draw.

The PBMCs were collected applying the standard Ficoll-Paque PLUS isolation procedure (GE Healthcare Life Sciences, Piscataway, NJ, USA), within 1 h of blood draw. PBMCs were aliquoted into 1.5 ml Eppendorf tubes (Eppendorf, Hamburg, Germany) and kept frozen at −80 °C until assayed.

Total RNA was extracted from PBMCs by use of TRIzol reagent (Life Technologies, Life Technologies Europe, Naerum, Denmark). RNA quality and quantification was measured spectrophotometrically using NanoDrop (NanoDrop Technologies, Wilmington, DE, USA) spectrophotometer and software applying the 260/280 and 260/230 ratio algorithms. cDNA was synthesized from RNA with a High Capacity dDNA Reverse Transcription Kit (Life Technologies). The cDNA was subjected to quantitative real-time PCR using the ViiA 7 Real-Time PCR System (Life Technologies) with TaqMan PCR Master Mix and using TaqMan gene expression probes (Life Technologies): Hs00241738_m1 (*ANK3*), Hs00167681_m1 (*CACNA1C*), Hs00996727_m1 (*RASGRP1*), Hs00971557_m1 (*KLF12*), Hs02718934_s1 (brain-derived neurotrophic factor), Hs00160298_m1 (*POLG*), Hs00963643_m1 (*PDE4B*), Hs00833126_g1 (*MAPK6*), Hs01652468_g1 (*PGAM1*), Hs00953724_m1 (*ADARB1*), Hs00159343_m1 (*NUDT1*), Hs00221478_m1 (*NDUFV2*), Hs01922715_s1 (*GPER1*), Hs00174860_m1 (*ESR1*), Hs01100353_m1 (*ESR2*), Hs00916521_m1 (*SP1*), Hs00162095_m1 (*SP4*), Hs01060665_g1 (*ACTB*), Hs01104728_m1 (*ABL1*), Hs02758991_g1 (*GAPDH*), Hs00188166_m1 (*SDHA*), Hs01047719_m1 (*GSK3B*), Hs00249899_m1 (*OGG1*). CACNA1C and brain-derived neurotrophic factor mRNA was undetectable in majority of the cases and were not included in further analyses. Samples were run in triplicate in each assay with laboratory personnel blinded to the clinical status of participants.

A set of three genes, the beta-actin (*ACTB*) gene, the C-Abl Oncogene 1 (*ABL1*) gene and the succinate dehydrogenase complex, subunit A, flavoprotein (Fp) (*SDHA*) gene, were used as candidate reference genes for normalization as these have been used in previous studies.^7, 8, 32^ The stability of candidate reference genes was assessed using the NormFinder software.^33^ The combination of *SDHA* and *ACTB* exhibited the highest stability in comparisons between bipolar disorder patients and healthy control subjects (*SDHA+ACTB* stability level of 0.002) and the combination of *ACTB* and *ABL1* demonstrated highest stability in comparisons between affective states within bipolar disorder patients (*ACTB+ABL1* stability level of 0.004). Semi-quantitative mRNA levels, assessed by cycle threshold (CT) were thus expressed relative to the mean values of *SDHA* and *ACTB* combined and mean values of *ACTB* and *ABL1* combined in respective comparisons. The ΔC_T_=C_T_ (each gene)−C_T_ (reference genes) was calculated for each sample and relative levels of expression were determined using the comparative C_T_ method,^34^ calculated by 2^−^^ΔCT^.

In addition, standard clinical chemistry parameters were analyzed, including fasting blood glucose and fasting lipid parameters.

### Statistics

Independent *t*-tests were used to test differences in age between healthy control subjects and bipolar disorder patients, and the chi-squared test was used to examine the differences in categorical demographic and clinical variables.

First, analyzing the full sample, comparisons of mRNA levels of all investigated genes between bipolar disorder patients and healthy control subjects were assessed in a two-level linear mixed effects model, accommodating both variation of the outcome variables within subjects (intra-individual variation) and between subjects (inter-individual variation). Level one represented repeated measures of mRNA levels as main effects and level two represented between-subject variation. A random intercept was included to accommodate correlations in the outcome variables over time within each participant and analysis was adjusted for age and gender. Values are expressed as the regression slope, b. All the analyses were conducted with individual mRNA levels as main effects. Assumptions of independence of errors, homoscedasticity and normality were met.

Similar mixed model analysis was performed investigating differences between affective states in bipolar disorder patients. Bonferroni correction was applied to control for multiple testing, resulting in a significance level of 0.05/19=0.0026. Genes for which mRNA expression differed between groups with a *P*-value of 0.05 or less were considered in the next step for building an exploratory abbreviated composite gene expression score.

For the calculation of a composite gene expression score, a split sample design was used, similar to the strategy described by Kato *et al.*,^8^ with the total sample randomly split into two equal-sized samples consisting of equal distributions of bipolar disorder patients and healthy control subjects. In the first sample (sample 1), mRNA expression levels of all the investigated genes were entered as covariates together with age and gender in a generalized linear mixed model specifying the repeated measures within participants and the intercept as random covariates and the binomial outcomes bipolar disorder patient vs healthy control and depressed state vs euthymic state and manic state vs euthymic state, respectively. A second abbreviated model including only the genes for which mRNA expression differed between groups with a *P*-value of 0.05 or less in the primary analysis step was additionally conducted. In these models, mRNA expression levels were centered around the grand mean of mRNA levels divided by the standard deviation, to assign more weight to markers with narrower confidence intervals and to make models across samples easily comparable.^35^ A composite gene expression score (*P*) signifying the probability of group membership was constructed using the formula:


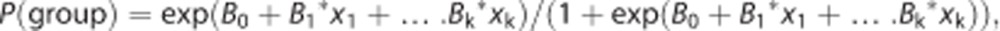


where *B*_0_ is a constant and *B*_1_ … *B*_k_ represent model coefficients and *x*_1_ … *x*_k_ are individual values of the predictor variables (mRNA expression level of each gene, age and gender) entered into the generalized linear mixed model.

The composite score was tested in split samples (sample 1 and sample 2) and the models were compared by receiver-operating characteristics (ROC) analysis.^36^ Assigning a cutoff on the constructed composite gene expression score based on the ROC analysis, sensitivity and specificity was calculated, focusing on obtaining the highest level of both measures. Finally, the accuracy of the composite gene expression score as a diagnostic test was assessed by calculating the likelihood ratios, which represent the probability of the test result in patients with a given disease to the probability of the same test result in patients without the disease^37^ and are stable to the prevalence of the disease. The positive likelihood ratio (LR [+]) was calculated as (sensitivity/1−specificity) and the negative likelihood ratio (LR [−]) was calculated as (1−sensitivity/specificity).

The statistical analysis was conducted with SPSS, version 22.0 (IBM, New York, NY, USA).

## Results

Clinical and demographic characteristics of the total study population are described in Table 1A and split samples characteristics are described in Supplementary Table 1S. Briefly, there were no overall statistically significant differences between bipolar disorder patients and healthy control subjects with regard to age, gender distribution, educational level or body mass index. All the participants were Caucasian and outpatients at the time of inclusion. Number of samples obtained and symptom severity at the time of assessment are presented in Table 1B.

### mRNA expression levels in bipolar disorder patients and healthy controls in the total sample

**Figure 1:**
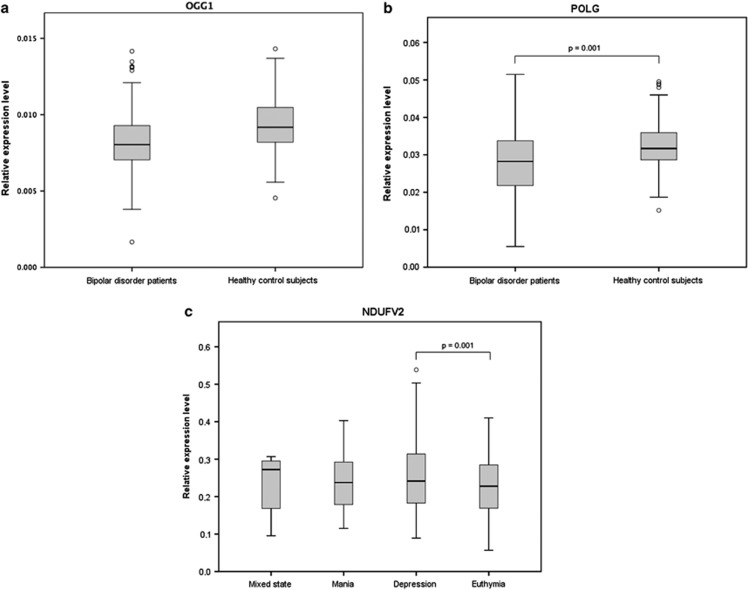
Boxplots of mRNA expression levels. *P*-values represent Bonferroni corrected significance levels in mixed model analysis adjusted for age and gender. Circles represent values outside the first or third quartile of more than 1.5 times the interquartile range. (**a** and **b**) *OGG1* and *POLG* gene expression were downregulated in bipolar disorder patients compared with healthy control subjects. (**c**) *NDUFV2* gene expression was upregulated in a depressed state compared with a euthymic state in bipolar disorder patients. mRNA, messenger RNA.

Adjusted for age and gender, mRNA levels of KLF12 (*b*=−0.0122, 95% CI: −0.0244 to −0.0004), POLG (*b*=−0.0034, 95% CI: −0.0059 to −0.0009), OGG1 (*b*=−0.0012, 95% CI: −0.0018 to −0.0005) and GSK3B (*b*=−0.0071, 95% CI: −0.0140 to −0.0002) were downregulated in bipolar disorder patients overall, whereas PGAM1 (*b*=0.0070, 95% CI: 0.0009 to 0.1310) was upregulated compared with healthy control subjects (all *P*<0.05; Table 2). After Bonferroni correction, POLG (*P*=0.001) and OGG1 (*P*=0.001) remained significantly downregulated in bipolar disorder patients (Figures 1a and b). In *post hoc* exploratory analysis, mRNA levels of both POLG and OGG1 remained downregulated when further adjusting for body mass index, smoking status and alcohol use (Supplementary Results).

In comparisons between affective states within bipolar disorder patients, also adjusted for age and gender, mRNA levels of *NDUFV2* (*b*=0.0414, 95% CI: 0.0170 to 0.0658), *ESR2* (*b*=0.0007, 95% CI: 0.0000 to 0.0014), *SP1* (*b*=0.0116, 95% CI: 0.0004 to 0.0229) and *NUDT1* (*b*=0.0043, 95% CI: 0.0009 to 0.0077) were upregulated in a depressed state compared with a euthymic state. *NDUFV2* (*b*=0.0354, 95% CI: 0.0007 to 0.0701) was additionally upregulated in a manic state compared with a euthymic state (Table 2). Only *NDUFV2* (*P*=0.001) upregulation in a depressed state remained statistically significant after Bonferroni correction (Figure 1c).

### mRNA expression as a composite gene expression score

#### Discrimination between bipolar disorder patients and healthy control subjects

**Figure 2:**
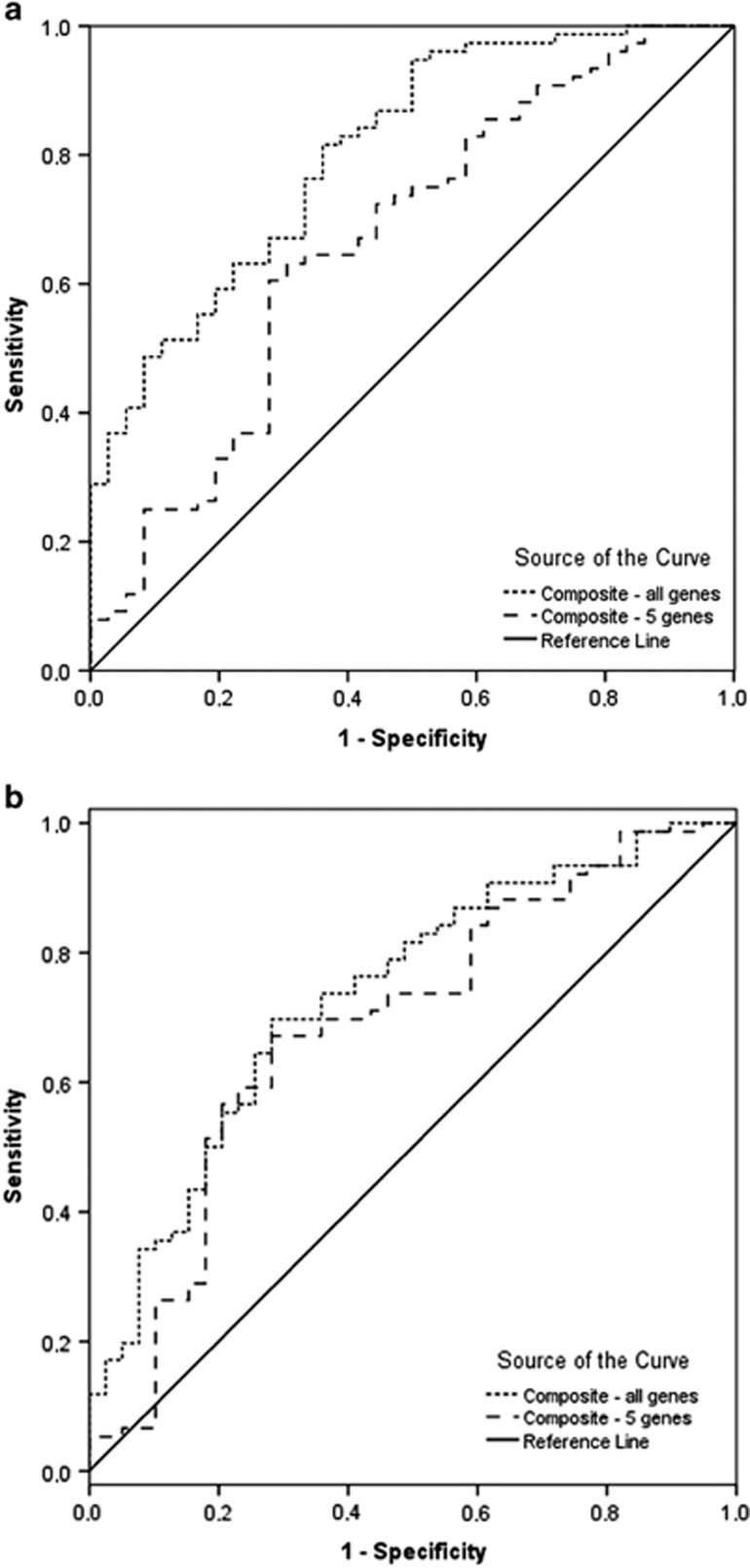
ROC curves of the composite gene expression measure as discriminant function between bipolar disorder patients and healthy control subjects. The composite measure based on all the 19 genes was superior to that based on the five genes more closely associated with a bipolar disorder diagnosis (*P*<0.05) in primary mixed model analysis. The discriminant capacity of both measures was higher in sample 1 (**a**) compared with sample 2 (**b**). ROC, receiver-operating characteristic.

Applying the full composite gene expression score on sample 1, the area under the ROC curve was 0.806 (95% CI: 0.721 to 0.891, *P*<0.0001; Table 3). Applying a composite score on the first sample based on the five genes identified in the primary analysis step with a *P*-value of 0.05 or less, the AUC of the ROC curve was 0.666 (95% CI: 0.554 to 0.777, *P*=0.005; Figure 2a), corresponding with an inferior discriminant capacity compared with the full composite score. Setting a cutoff of 0.5 on the full composite gene expression score, bipolar disorder patients and healthy control subjects were discriminated with a sensitivity of 78% and a specificity of 60% (*χ*^2^=17.24, *P*<0.0001). The corresponding values for the abbreviated model were 63 and 60% (*χ*^2^=7.35, *P*=0.007). Repeating the ROC analyses on the second sample (sample 2), AUCs of the ROC curves were 0.734 (95% CI: 0.638 to 0.831, *P*<0.0001) and 0.687 (95% CI: 0.580 to 0.793, *P*=0.001) for the full composite score and the abbreviated score, respectively (Figure 2b). In this sample, bipolar disorder patients and healthy control subjects were discriminated with a sensitivity and specificity of 62 and 75% (*χ*^2^=14.65, *P*<0.0001) applying the full composite gene expression score, with a cutoff of 0.5.

The calculated positive and negative likelihood ratios using the full gene set were 2.0 and 0.37 in sample 1, respectively and 2.5 and 0.50 in sample 2, respectively. This indicated a relatively small shift in probability of a correct diagnosis using the full gene set. For the abbreviated gene score, the positive and negative likelihood ratios were 1.6 and 0.6 in sample 1 and 3.0 and 0.5 in sample 2, corresponding with an overall less accurate test for the abbreviated set.

#### Discrimination between affective states in bipolar disorder patients

The full composite gene expression measure discriminated between depressed and euthymic states with a sensitivity of 91% and a specificity of 75% (*χ*^2^=19.06, *P*<0.0001) in sample 1 and 60 and 60% (*χ*^2^=2.97, *P*=0.09) in sample 2, respectively, assigning a cutoff score of 0.7 (Table 3). This surpassed the effectiveness of the abbreviated gene expression measure of the four genes identified in the initial steps (Table 3). Discriminating between manic states and euthymic states in the first sample, the full measure demonstrated a sensitivity of 92% and a specificity of 66% (*χ*^2^=12.42, *P*<0.0001), whereas the corresponding values for the abbreviated set were 0 and 97%, respectively. In sample 2, the full expression score discriminated between manic and euthymic patients with a sensitivity of 45% and a specificity of 65% (*χ*^2^=0.420, *P*=0.5).

In exploratory correlation analyses, the potential correlation between medication classes and scores on the full composite gene expression measure discriminating between bipolar disorder patients in a current affective state and a euthymic state was investigated using Pearson product–moment correlation. In these analyses, only antipsychotic use was moderately correlated with lower probability of bipolar disorder patients being in a manic compared with a euthymic state (*r*(39)=−0.58, *P*<0.0001) with no correlation between either lithium, anticonvulsant or antidepressant treatment and composite gene expression measure scores.

## Discussion

In the present study, we investigated the expression of 19 candidate biomarker genes in the PBMCs in rapid cycling bipolar disorder patients longitudinally across different affective states and as repeated measures in healthy control subjects. We found downregulation of two genes, *POLG* and *OGG1*, in bipolar disorder patients compared with healthy control subjects after correction for multiple testing and adjusting for possible confounders. In comparisons between affective states, we found increased *NDUFV2* expression in a depressed state compared with a euthymic state. Further, a composite gene expression measure was constructed on the basis of individual gene expression levels and its discriminant capacity validated in an independent cohort. The composite gene expression measure for discrimination between bipolar disorder patients and healthy control subjects based on 19 genes generated an area under the ROC curve of 0.81 (*P*<0.0001) in sample 1, which was replicated with a value of 0.73 (*P*<0.0001) in sample 2. This corresponds with a moderately accurate test^38^ and surpassed that based on an abbreviated set of genes, identified by being more closely associated with a bipolar diagnosis.

*OGG1* expression dysregulation is a novel finding in bipolar disorder. *OGG1* encodes the 8-oxoguanine DNA glycosylase, the primary enzyme responsible for the excision of 8-oxoguanine (8-oxodG), an oxidated DNA guanine nucleoside resulting from exposure to reactive oxygen species. In knockout mice, it has consistently been demonstrated that lacking an *OGG1* repair system leads to increased accumulation of oxidative DNA lesions.^39^ Animal studies further suggest that *OGG1* deficiency could increase susceptibility to neurodegeneration under conditions of increased oxidative stress.^40^ Accumulation of oxidatively generated DNA damage has been associated with cardiovascular disease^41^ and diabetes,^42^ which are also associated with bipolar disorder. Further, oxidatively generated DNA damage may contribute to a shortened lifespan,^43^ also observed in bipolar disorder.^44^ Recently, we showed high levels of oxidatively generated damage to DNA in this cohort, for the first time demonstrating elevated levels of urinary excreted 8-oxodG in bipolar disorder patients through all affective phases (hypomania/mania, depression and euthymia) compared with healthy control subjects.^21^ It is thus possible that the *OGG1* downregulation identified in the present study may lead to accumulation of oxidative DNA lesions and increased total levels of oxidatively generated damage to DNA, reflected by the observed high levels of 8-oxodG that was previously reported.^21^ The relationship between base excision repair and urinary excretion of oxidatively damaged nucleosides, however, is complex and incompletely understood,^45^ and a causal relationship cannot be established on the basis of our findings.

*POLG* downregulation in bipolar disorder has previously been demonstrated in lymphoblastoid cells;^8^ however, we believe our study is the first to demonstrate *POLG* downregulation in PBMCs of bipolar disorder patients. Mutations in the *POLG* gene encoding the catalytic gamma subunit of mitochondrial DNA polymerase cause multiple deletions or depletion of mitochondrial DNA alone or in combination and are associated with mitochondrial diseases with a wide range of clinical manifestations.^46^ Interestingly, transgenic mice with brain-specific expression of mutant *POLG* exhibit a phenotype resembling bipolar disorder with antidepressant-induced mania-like behavior and periodic activity related to estrous cycle in female animals.^47^ The mood-stabilizer valproate was additionally demonstrated to alter *POLG* gene expression *in vitro*.^48^ Mitochondrial dysfunction has been linked with the pathophysiology of bipolar disorder^49^ and clinically, high rates of comorbidity between mitochondrial disorders and bipolar disorder, with psychiatric symptoms often being the prominent and presenting feature of mitochondrial disorders.^50^ Mice expressing a proof-reading-deficient version of *POLG* display features of accelerated aging and a shortened lifespan^51^ as well as gender-dependent hypertension,^52^ which is noteworthy considering that bipolar disorder is associated with cellular signs of accelerated aging^53^ and a high occurrence of cardiovascular comorbidity.^54^ Our finding of aberrant gene expression of *POLG* lends further support to a role for *POLG* in bipolar disorder pathophysiology.

*NDUFV2* expression has not previously been described in PBMCs of bipolar disorder patients and state-related alterations of *NDUFV2* specifically have not been investigated. The nuclear gene *NDUFV2* encodes the NADH dehydrogenase (ubiquinone) flavoprotein 2a subunit of the mitochondrial complex I, which is involved in oxidative phosphorylation and proton transport. Several lines of evidence implicate *NDUFV2* in bipolar disorder. *NDUFV2* is located at 18p11, a reported susceptibility locus for bipolar disorder^55^ and polymorphisms in the upstream region of *NDUFV2* have also been associated with bipolar disorder.^56, 57^ Further, upregulation of NDUFV2 expression in postmortem brain samples from bipolar disorder patients compared with healthy control subjects have been described.^58^ Three studies have investigated *NDUFV2* expression in lymphoblastoid cell lines, with inconsistent findings of both downregulation of *NDUFV2* in bipolar I patients^20, 57^ and upregulation in bipolar II patients,^20^ whereas one study found no differences between bipolar disorder patients and healthy control subjects.^59^ One possible reason for the discrepant findings may be that the previous studies included patients in various affective states, not having characterized the affective state of participants. Our finding of upregulation of *NDUFV2* expression in a depressed state compared with a euthymic state could indicate that alterations of *NDUFV2* expression are state related, suggesting a possible role for *NDUFV2* as a state biomarker.

Of note, our finding of upregulation of just one gene in primary analysis, the *PGAM1*, in bipolar disorder patients compared with healthy control subjects mirrored previous findings in lymphoblastoid cells.^19^

Our investigation of a composite gene expression measure yielded somewhat promising results. The likelihood ratios for the composite gene expression scores were overall modest (<3 and >0.3 for LR [+] and LR [−], respectively), which indicates a relatively small effect on posttest probability corresponding to a limited value as a diagnostic test by itself. This indicated a relatively small shift in the probability of a correct diagnosis using the full gene set, however, not excluding a somewhat useful property for the test in certain situations. Choosing a cutoff on the composite measure that placed equal value on sensitivity and specificity, a sensitivity of 78% and specificity of 60% was obtained in the first sample with values of 62 and 75% in the second sample. Although the values obtained in the first sample are likely inflated by nature, the sensitivity and specificity values obtained in the second sample are comparable to tests in the other areas of medicine such as the prostate-specific antigen test for prostate cancer (sensitivity of 21% and specificity of 91%)^60^ and the MagStream HemSp fecal immunochemical test for the detection of colonic neoplasms (sensitivity of 23.2% and specificity of 87.6%).^61^ The superior discriminant capacity of the composite measure based on the full set of genes as compared with the abbreviated set is indicative of the importance of including several individual potential biomarkers, which by themselves may contribute only discretely. Further, it is possible that the additional inclusion of laboratory values on a protein level, that is, inflammatory markers and markers of oxidative stress could increase the strength of the composite measure as a useful diagnostic test.

Our study benefitted from several methodological aspects. We applied careful standardization of blood sampling conditions, adhering to a short interval during the morning and obtaining samples in a fasting state. We further ensured blinding of laboratory staff to participant status and, crucially, we measured the expression of several candidate reference genes and evaluated their stability in contrast to previous studies^7, 8, 12^ that included only one reference gene, which is not recommended.^62^ We further used a split sample design in the evaluation of the full composite gene expression measure, allowing for testing this in independent samples. Finally, we assessed gene expression prospectively in patients during depressive, manic and euthymic states, which no other study has done.

Some limitations apply to the present study. First, the sample size was relatively small, and because not all patients experienced episodes of all polarities, the amount of between-subject variation relative to within-subject variation was therefore relatively large. Future studies should include larger sample sizes that would potentially allow for strict within-subject analyses and a further exploration of biomarker candidates to function in a personalized manner. Second, our findings primarily relate to mitochondrial function, which is influenced by lithium, mood-stabilizers and antipsychotics,^63^ although the direction and nature of the association is not uniform and knowledge about the effect of medication on gene expression in peripheral blood is limited. As the included bipolar disorder patients were medicated, we cannot entirely rule out the possibility that differences in the gene expression between bipolar disorder and healthy control subjects were due to, or at least partially explained by, an effect of medication. The effect of medication on *OGG1* and *POLG* expression in bipolar disorder patients *in vivo* has not previously been investigated. *POLG* expression has been demonstrated to increase *in vitro* after valproate administration,^48^ potentially indicating, that the downregulation we observed was not due to mood-stabilizing medication. Findings of *NDUFV2* expression in lymphoblastoid cells that are likely free of influence of medication are inconsistent, showing both elevated and decreased *NDUFV2* gene expression in bipolar disorder patients compared with healthy control subjects,^20^ and one small study (*n*=4) found *NDUFV2* upregulated after the administration of valproate but unaltered after lithium administration.^20^ The effect of medication on *NDUFV2* expression is thus unclear, not giving specific indication as to the potential influence of medication on the finding of upregulated *NDUFV2* expression in a depressed state compared with a euthymic state in bipolar disorder patients in the present study.

In comparisons between affective states within bipolar disorder patients, however, medication likely did not influence results to a large degree, as majority of the patients did not change medication during the study. Along these lines, exploratory analyses did not indicate an influence of medication on the composite gene expression measure discriminating between affective states in bipolar disorder patients. In future studies, it will be valuable to study unmedicated patients in comparison with healthy control subjects. However, for comparisons between affective states, it is likely not feasible to study unmedicated rapid cycling bipolar disorder patients longitudinally, due to the severity of illness. Third, the abbreviated composite gene expression measure was developed in the entire sample and the split sample design, therefore, did not constitute a genuine replication in the abbreviated gene set. Finally, the mean duration of illness for the bipolar disorder patients was relatively long and because neurobiological mechanisms potentially differ depending on the illness stage,^64^ findings may not be generalizable to all the bipolar disorder patients.

An issue that applies to studies investigating gene expression in peripheral blood in general pertains to the relationship between gene expression in the brain and that of peripheral blood. Although it is unclear to what extent peripheral blood gene expression patterns reflect those of the brain,^65^ peripheral blood cells express a large proportion of the genes in the human genome^66^ and a significant proportion of SNP-expression relationships are conserved between the brain and peripheral blood lymphocytes.^67^ The peripheral blood transcriptome may thus reflect system-wide biology and as such be a relevant tissue source for biomarker candidates. However, it is not clear whether it is a relevant surrogate tissue in relation to the brain.^68^

Candidate gene expression markers for the present study were selected *a priori* using a hypothesis-driven and transparent approach on the basis of previous gene expression findings and current hypotheses regarding the pathophysiology of bipolar disorder. The method involved combining potential biomarkers within multiple pathways in an effort to capture some of the complexity involved in the pathophysiology of bipolar disorder. Biomarker discovery in neurodegenerative^69^ and medical disorders such as cancer,^70^ diabetes and cardiovascular disease^71^ have used both a hypothesis-driven and a hypothesis-free, data-driven approach. Although facing the challenge of identifying clinically meaningful biomarkers,^72^ a systems-based approach integrating hypothesis-free biomarker discovery and networks is, by itself, likely superior, given its ability to better interrogate the multivariate and combinatorial characteristics of cellular networks, that are implicated in complex disorders,^73^ and a combination of both data-driven methods and knowledge-based hypotheses-driven methods appear promising.^74^ In this regard, our strictly hypothesis-driven approach could be considered a limitation.

In conclusion, our results suggest a potential for a composite gene expression measure as a diagnostic biomarker of bipolar disorder. In addition, we demonstrated aberrant regulation of the *POLG*, *NDUFV2* and, for the first time, the *OGG1* gene, pointing to disturbances within mitochondrial function and DNA damage repair mechanisms as pathophysiological mechanisms in bipolar disorder. The findings need replication in larger samples.

## Figures and Tables

**Table 1:** Demographic and clinical characteristics of study participants

*A. Characteristics at inclusion*	*Bipolar disorder patients*	*Healthy control subjects*	*Statistic*	P*-value*
*N*	37	40		
Age (years)	40.9±12.3	36.3±12.5	*t*=1.828	0.1
Gender (female–male)	25–12	23–17	*χ*^2^=0.830	0.3
Education (years total)	16.1±3.0	16.4±2.3	*t*=0.608	0.5
Body mass index	24.6±3.6	24.9±3.9	*t*=0.353	0.7
Duration of illness (years)	21.2±13.0 (2–56)			
Bipolar I (%)	22 (59.5)			
Bipolar II (%)	15 (40.5)			
Number of depressive episodes	16.2±15.4			
Number of hypomanic episodes	16.5±19.1			
Number of manic episodes	3.2±7.1			
Number of hospitalizations	10.2±19.5			
Lithium treatment (%)	15 (40.5)			
Anticonvulsant treatment (%)	27 (73.0)			
Antipsychotic treatment (%)	27 (73.0)			
SSRI treatment (%)	8 (21.6)			
Newer antidepressant treatment (%)	2 (5.4)			
Older antidepressant treatment (%)	2 (5.4)			

*B. Number of samples and symptom severity of participants at time of assessment*					
	*Samples from healthy control subjects* *(*N=*80)*	*Samples from bipolar disorder patients* *(*N=*168)*			
		*Euthymic*	*Depressive*	*Manic/hypomanic*^a^	*Mixed state*
		*(*N=*75)*	*(*N=*63*)	*(*N=*24)*	*(*N=*6)*
HAMD-17	0.6±0.9	3.7±1.9	15.5±5.1	3.4±2.6	10.2±1.8
YMRS	0.4±0.8	1.0±1.7	0.9±1.4	15.3±4.3	11.2±2.8

Abbreviations: HAMD-17, Hamilton rating scale, 17 items; SSRI, selective serotonergic reuptake inhibitor; YMRS, Young mania rating scale.

a Manic patients, *n*=19/hypomanic patients, *n*=5.

Data are expressed as mean (±s.d.) or *n* (%). Data are expressed as mean ±s.d. *N* represents number of samples. Values are presented as raw values, unadjusted for repeated measures.

**Table 2:** Gene expression levels in bipolar disorder patients compared with healthy control subjects and between affective states in bipolar disorder patients

*Gene*	*BD vs HC*^a^			*Within BD*^b^								
	*BD vs HC (ref)*			*DEP vs EU (ref)*			*MAN vs EU (ref)*			*MAN vs DEP (ref)*		
	b	*95% CI*		b	*95% CI*		b	*95% CI*		b	*95% CI*	
		*Min*	*Max*		*Min*	*Max*		*Min*	*Max*		*Min*	*Max*
*NDUFV2*	0.0014	−0.0073	0.0102	0.0414*	**0.0170**	**0.0658**	**0.0354**^**†**^	**0.0007**	**0.0701**	−0.0063	−0.0045	0.0324
*ESR1*	−0.0006	−0.0024	0.0011	0.0031	−0.0005	0.0067	0.0021	−0.0030	0.0072	−0.0010	−0.0061	0.0040
*ESR2*	0.0000	−0.0002	0.0004	**0.0007**^**†**^	**0.0000**	**0.0014**	0.0007	−0.0002	0.0016	0.0001	−0.0010	0.0011
*KLF12*	−**0.0122**^**†**^	−**0.0244**	−**0.0004**	0.0077	−0.0319	0.0473	0.0015	−0.0550	0.0581	−0.0062	−0.0624	0.0501
*SP4*	−0.0024	−0.0066	0.0017	0.0001	−0.0015	0.0112	−0.0033	−0.0193	0.0128	−0.0056	−0.0238	0.0127
*SP1*	0.0025	−0.0016	0.0066	**0.0116**^**†**^	**0.0004**	**0.0229**	−0.0022	−0.0180	0.0136	−0.0112	−0.0292	0.0068
*PGAM1*	**0.0070**^**†**^	**0.0009**	**0.1310**	−0.0016	−0.0204	0.0172	0.0008	−0.2572	0.0273	−0.0007	−0.0305	0.0290
*ANK3*	−0.0002	−0.0025	0.0021	0.0030	−0.0019	0.0079	0.0014	−0.0056	0.0084	−0.0016	−0.0086	0.0054
*RASGRP1*	−0.0015	−0.0048	0.0018	0.0012	−0.0078	0.0103	0.0011	−0.0117	0.0140	−0.0025	−0.0171	0.0121
*AKT1*	−0.0032	−0.0153	0.0089	0.0062	−0.0187	0.0312	0.0120	−0.0234	0.0475	−0.0071	−0.0450	0.0358
*NUDT1*	0.0012	−0.0002	0.0026	**0.0043**^**†**^	**0.0009**	**0.0077**	0.0026	−0.0023	0.0074	0.0007	−0.0059	0.0074
*POLG*	−0.0034*	−**0.0059**	−**0.0009**	0.0025	−0.0074	0.0123	0.00160	−0.0123	0.0155	−0.0017	−0.0172	0.0138
*ADARB1*	−0.0010	−0.0020	0.0006	0.0015	−0.0018	0.0048	−0.0003	−0.0049	0.0043	−0.0023	−0.0077	0.0030
*OGG1*	−0.0012*	−**0.0018**	−**0.0005**	0.0009	−0.0020	0.0038	0.0006	−0.0034	0.0047	−0.0004	−0.0049	0.0040
*PDE4B*	0.0027^‡^	−0.0004	0.0058	0.0110	−0.0010	0.0230	0.0093	−0.0088	0.0254	−0.0027	−0.0197	0.0143
*GSK3B*	−**0.0071**^**†**^	−**0.0140**	−**0.0002**	0.0109	−0.0152	0.0370	0.0071	−0.0302	0.0445	−0.0037	−0.0408	0.0333
*APOE*	0.0000	0.0000	0.0001	0.0001	−0.0002	0.0003	−0.0001	−0.0005	0.0002	−0.0001	−0.0005	0.0002
*GPER1*	−0.0001	−0.0004	0.0002	0.0003	−0.0005	0.0011	−0.0006	−0.0017	0.0005	−0.0007	−0.0016	0.0001
*MAPK6*	0.0003	−0.0005	0.0012	0.0004	−0.0014	0.0021	0.0001	−0.0024	0.0026	−0.0001	−0.0042	0.0028

Abbreviations: BD, bipolar disorder; CI, confidence interval; DEP, depression; EU, euthymia; HC, healthy control; MAN, mania; ref, reference category.

a Gene expression normalized to mean expression of *ACTB* and *SDHA* combined.

b Gene expression normalized to mean expression of *ACTB* and *ABL* combined.

*P<0.001; ^†^*P*<0.05; ^‡^*P*<0.1. All the analyses were adjusted for age and gender; *b* represents slope. Values with a *P*-value of 0.05 or less are in bold.

**Table 3:** ROC analyses of composite gene expression scores in all comparisons.

*Comparison*	*Sample*	*Composite gene set*	*AUC*	*95% CI*		P*-value*	*Sensitivity (%)*	*Specificity (%)*
				*Min*	*Max*			
BD vs HC	1	All genes^a^	0.806	0.721	0.890	<0.0001	78	60
		5 genes^b^	0.666	0.554	0.777	0.005	63	60
	2	All genes	0.734	0.638	0.831	<0.0001	62	75
		5 genes^b^	0.687	0.580	0.793	0.001	59	80
								
DEP vs EU	1	All genes^c^	0.882	0.793	0.970	<0.0001	91	75
		4 genes^d^	0.620	0.479	0.761	0.104	67	44
	2	All genes	0.542	0.395	0.690	0.565	60	60
		4 genes^d^	0.649	0.515	0.783	0.043	73	42
								
MAN vs EU	1	All genes^e^	0.848	0.732	0.965	0.001	92	66
		4 genes^d^	0.524	0.357	0.690	0.807	0	97
	2	All genes	0.584	0.418	0.750	0.436	45	65
		4 genes^d^	0.661	0.498	0.823	0.136	9	89

AUC, area under the curve; BD, bipolar disorder; CI, confidence interval; DEP, depression; EU, euthymia; HC, healthy control; MAN, mania; ROC, receiver-operating characteristic.

a Cutoff score 0.5.

b KLF12, PGAM1, POLG, OGG1 and GSK3B.

c Cutoff score 0.7.

d NDUFV2, ESR2, SP1 and NUDT1.

e Cutoff score 0.01.

## Notes

LVK has within the preceding 3 years been a consultant for Lundbeck and AstraZeneca. MV has been a consultant for Lundbeck, AstraZeneca, Eli Lilly and Servier. The remaining authors declare no conflict of interest.
