# Supplementary material for: A composite peripheral blood gene expression measure as a potential diagnostic biomarker in bipolar disorder
Source: Transl Psychiatry. 2015 Aug 4;5(8):e614–. doi: 10.1038/tp.2015.110 (PMC4564565; doi:10.1038/tp.2015.110)
Supplement: Supplementary Information [file tp2015110x1.doc]

*A composite peripheral blood gene expression measure as a potential diagnostic biomarker in bipolar disorder*

Klaus Munkholm, M.D.

Lone Peijs

Maj Vinberg, M.D., Ph.d.

Lars Vedel Kessing, professor, M.D., DMSc

This material supplements but does not replace the content of the peer-reviewed paper published in Translational Psychiatry

***Supplementary information***

**Methods**

*Participants*

The majority of bipolar disorder patients received specialized treatment at the Mood Disorders Clinic, Psychiatric Center Copenhagen, Rigshospitalet, Copenhagen, Denmark. 73% of the patients did not change type of medication during the study despite alterations of affective state, owing to the mulitfaceted and highly specialized treatment received, including non-pharmacological interventions.

**Supplementary Table S1.** Characteristics of participants in split sample design.

|  | *Sample 1* | | *Statistic* | *Sample 2* | | *Statistic* |
| --- | --- | --- | --- | --- | --- | --- |
|  | *BD* | *HC* |  | *BD* | *HC* |  |
| N | 19 | 20 |  | 18 | 20 |  |
| Gender (m-f) | 6-13 | 9-11 | P=0.4 | 6-12 | 8-12 | P=0.7 |
| Age | 38.84±11.64 | 39.69±13.00 | P=0.8 | 43.13±12.87 | 32.98±11.28 | P=0.01 |

BD: bipolar disorder patients; HC: healthy control subjects; m-f: male-female. Test statistics used were Pearson Chi-square Tests for comparison of gender distribution by group and independent T-test for comparison of age between groups.

**Results**

Blood samples were on average collected from bipolar patients 3.4±1.7 (1-10) times during the study. Samples were obtained during euthymia in all but three patients (mean 2.0±1.3 (0-6)), major depression in 26 patients (mean 1.7±1.7 (0-5)), mania/hypomania in 11 patients (mean 0.7±1.2 (0-5)) and in a mixed state in a total of 6 patients (mean 0.2±0.4 (0-1)).

In post-hoc linear mixed model analysis further adjusting significant findings for smoking status, BMI and alcohol consumption as covariates, mRNA levels of both *POLG* (b = -0.0040, 95% CI: -0.0070 to -0.0010, p = 0.01) and OGG1 (b = -0.0016, 95% CI: -0.0024 to -0.0008, p<0.0001) remained downregulated compared with healthy control subjects.
